# Cardiac fibrosis in the elderly, normotensive athlete: case report and review of the literature

**DOI:** 10.1186/1746-1596-3-12

**Published:** 2008-03-19

**Authors:** Shaheen E Lakhan, Lindsey Harle

**Affiliations:** 1 Global Neuroscience Initiative Foundation, Los Angeles, CA, USA

## Abstract

**Background:**

Cardiac fibrosis occurs with normal aging, but the extent of this process and its effect on cardiac function is unknown. Fibrosis in the nonhypertensive elderly patient is thought to be due to decreased degradation, and not increased deposition, of collagen. The cause of this decreased degradation is unknown. Athletes commonly develop cardiac hypertrophy, and recent evidence has linked long-term physical activity to the development of interstitial myocardial fibrosis. Whether this exercise-induced fibrosis occurs regularly, or only in genetically predisposed individuals, is unknown.

**Case presentation:**

We present the case of an elderly, nonhypertensive athlete who died suddenly of sepsis. Autopsy demonstrated foci of fibrosis throughout the right and left ventricle and significant narrowing of the left ventricular cavity. The findings may be secondary to aging, athletic activity or an undiagnosed medical condition.

**Conclusion:**

The true incidence and importance of age- and exercise-associated myocardial fibrosis is an area for future research.

## Background

Myocardial fibrosis occurs in a number of pathological processes, most commonly hypertension. Other disease states capable of producing cardiac fibrosis include hypereosinophilia, scleroderma, sarcoidosis, radiation and drug effects, viral myocarditis and inherited genetic mutations. Normal aging is also associated with a certain degree of interstitial fibrosis, but the degree of this nonpathological fibrosis is yet to be determined. Animal models of aging have shown alterations in collagen deposition [1], and studies involving human subjects and post-mortem examinations have documented increased fibrosis in the conduction system [2] and atria [3] with aging, but the true incidence of global myocardial fibrosis that can be expected in a healthy, normotensive elderly patient is not known.

## Case presentation

The patient was a 73-year-old healthy African American female with no significant medical history. Notably, she was an avid athlete and power lifter, participated regularly in aerobic activity and traveled frequently. She presented initially with a 2-day history of sore throat and fever and acute onset of mental status changes. Upon arrival at the hospital she was found to be febrile, hypertensive, tachycardic and tachypneic and to have an elevated white blood cell count of 15.2 K/ul. Physical examination revealed no obvious source of infection, and EKG showed tachycardia but otherwise normal electrical activity. The patient rapidly decompensated and died despite resuscitative efforts. Pre-mortem blood samples were later found to be positive for group A beta-hemolytic Streptococci. Postmortem examination of the patient's medical records revealed no significant disease history. She had a favorable cholesterol profile, with total cholesterol of 183 mg/dl, triglycerides 66, LDL 98 and HDL 72. There was no history of hypertension or evidence of cardiac disease and no history of eosinophilia.

**Figure 1:**
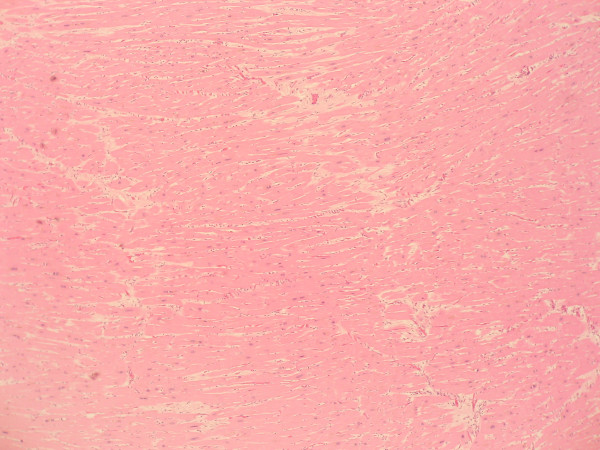
**Histological section of myocardium (low magnification).** Some areas showed only myocyte hypertrophy.

**Figure 2:**
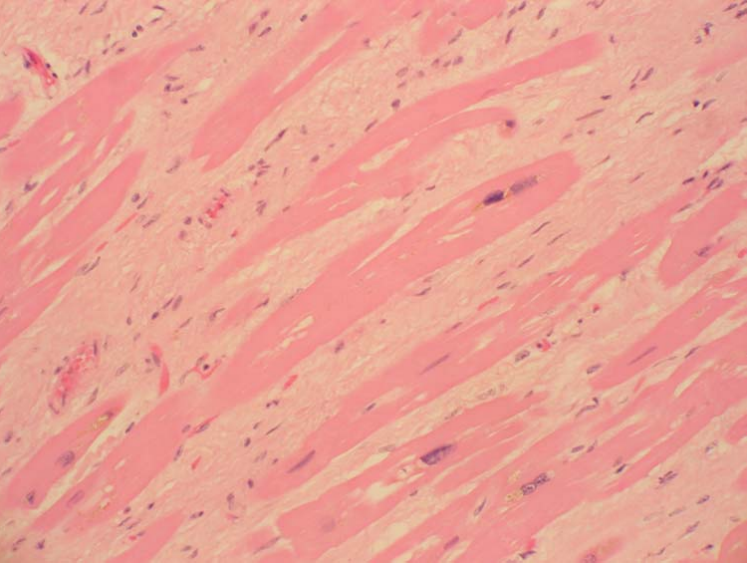
**Histological section of myocardium with areas of patchy interstitial fibrosis associated with myocyte hypertrophy (high magnification).** Fibrosis was present in both ventricular walls, and no single large focus of fibrosis was identified. Inflammation was minimal and the coronary arteries showed no atherosclerotic disease.

**Figure 3:**
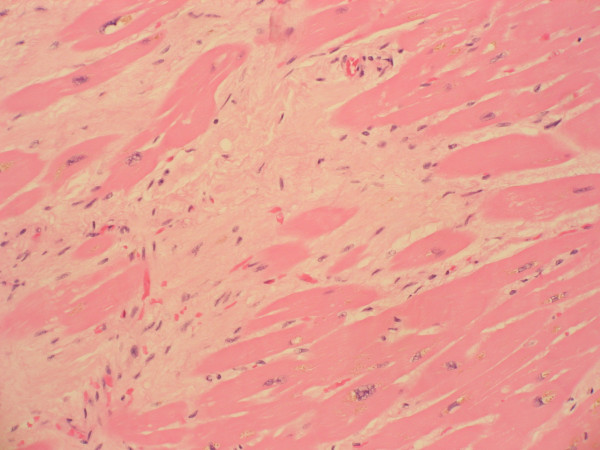
Histological section of myocardium with areas of patchy interstitial fibrosis associated with myocyte hypertrophy (high magnification).

Autopsy was performed and revealed no pathological processes acutely related to death, including no signs of localized infection as the source of bacteremia. The patient was found to have cardiomegaly with a cardiac weight of 365 grams, left ventricular wall thickness of 1.8 mm and markedly decreased chamber volume. Histological examination showed wide distribution of interstitial myocardial fibrosis (Figures 1, 2 and 3). Fibrosis was present throughout the right and left ventricles in an inconsistent pattern and was more prevalent in the endomyocardium. The largest foci of fibrosis measured approximately 4 mm in greatest diameter, and approximately 25% of the myocardium was involved by some degree of fibrosis. A single foci of inflammatory infiltrate was noted, but was not located near an area of fibrosis. Microfiber disarray and septal hypertrophy were absent. The coronary arteries were notably patent with minimal atherosclerosis.

## Discussion

While the cardiac fibrosis in this patient was not the immediate cause of death, the findings of a diffuse distribution of patchy fibrosis and myocardial hypertrophy in a patient with no history of hypertension, hyperlipidemia or cardiac disease are puzzling. Some degree of fibrosis is expected in the healthy elderly myocardium, but likely to a smaller degree than seen in the present case. Myocardial hypertrophy in the elderly is most commonly a response to reduced vascular compliance, but in the absence of hypertension the degree of ventricular wall thickening and loss of chamber volume seen in this patient would not be expected. This patient's history of athletic activity, including power lifting, would support the finding of myocardial hypertrophy. Athletes are known to have physiological cardiac hypertrophy, but this is not usually associated with the development of fibrosis or chamber narrowing.

Fibrosis within the myocardium may be due to a variety of different pathological processes, and the pattern of fibrosis provides clues as to the underlying cause. Hypertensive cardiac fibrosis is classically perivascular with infiltration into the surrounding interstitium. A single large focus of fibrosis is consistent with remote myocardial infarction; hypereosinophilia and idiopathic restrictive cardiomyopathy produce fibrosis ranging from patchy subendocardial disease to diffuse full-thickness involvement. Radiation-induced fibrosis is often more severe in the right ventricle.

### Aging and myocardial fibrosis

Changes associated with aging are difficult to separate from those due to diseases prevalent in the elderly, most notably hypertension. Histological changes seen in the non hypertensive aging heart include increased myocyte necrosis and apoptosis [4] and an altered deposition of types I and III collagen [1]. An autopsy study of 230 noncardiac patients demonstrated increased fibrosis and fat within the cardiac conduction system of elderly patients [2]. An age-related increase in right atrial fibrosis and decrease in nerve plexus population has also been demonstrated [3]. Burns [5] reported increased cardiac fibrosis, significantly more prominent in the posterior left ventricle, with aging. Klima [5] reported increased interstitial fibrosis related to aging that was independent of other cardiac diseases. A comparison of endomyocardial biopsies from elderly and young patients found increased cell diameter and nuclear area (a sign of increased protein production) and an increased deposition of lipid and lipofuscin with age [6].

While hypertensive patients show increased deposition of collagen, nonhypertensive aging patients develop increased interstitial collage levels due to decreased degradation [7,8]. Antihypertensive therapy prevents left ventricular fibrosis, which is secondary to pressure overload, but is ineffective in preventing right ventricular fibrosis [9], supporting the concept of disease-independent cardiac fibrosis in elderly patients.

### Biochemical mechanisms of myocardial fibrosis

Hypertensive cardiac fibrosis is related to the direct effects of pressure overload and to circulating hormones. Perivascular fibrosis and cardiac fibroblast activation correlate with increased activity of the renin-angiotensin-aldosterone system (RAAS) and elevated serum mineralocorticoids; this fibrosis is preventable with angiotensin-converting enzyme (ACE) inhibitors or spironolactone [10,11]. In heart failure, elevations in aldosterone and angiotensin II result from ischemia of the kidney and adrenal gland, as well as increased salt intake. Weber [12] reported local production of angiotensin II within the myocardium, suggesting a role for intrinsic myocardial activation of ACE secondary to ischemia.

The molecular activity of angiotensin II and adrenergic stimulation involve the extracellular-signal-related kinases (ERK1/2), p38 and Jun N-terminal kinase (JNK) [13,14], leading to fibroblast activation. Inflammation is also involved in hypertensive collagen deposition; Nicoletti [14] emphasized the role of inflammation in the development of cardiac fibrosis. Angiotensin II activates monocytes [15]. Infiltration of inflammatory cells has been documented in animal models of the hypertensive heart [14,16,17]. The inflammatory infiltrate is initially perivascular and later spreads interstitially, following the same pattern as that of collagen deposition [16]. Myocardial remodeling in the development of congestive heart failure has also been attributed to reactive oxygen species (ROS) production by the mitochondrial, xanthine oxidase, nitric oxide synthetase and NADPH oxidase pathways [18,19].

Myocardial fibrosis that occurs with normal aging should not be dependent upon the RAAS or inflammatory mediators, as neither of these systems are activated in the healthy elderly patient. Even in the absence of overt hypertension, arterial vascular walls loose compliance with age, resulting in some degree of pressure overload with normal aging. Whether this age-related pressure overload is severe enough to cause cardiac ischemia and fibrosis is unknown. Aging rats show increased left ventricular weight, extracellular collagen and collagen cross-linking, and decreased mRNA collagen expression [20]. This supports the theory that an age-related increase in cardiac fibrosis is not secondary to increased production, but instead is due to decreased destruction of existing collagen.

### Exercise induced changes in the myocardium

It is well documented that athletes frequently develop left ventricular hypertrophy [21], but this is not thought to be associated with fibrosis, as it regresses upon cessation of physical activity [22]. Lindsay and Dunn [23] questioned the validity of the common theory that exercise-induced hypertrophy is entirely physiological, based on the fact that veteran athletes often do not experience complete resolution of ventricular hypertrophy [24,25], suggesting a mechanism other than pure myocyte hypertrophy. Athletes are prone to the development of EKG abnormalities [25]. Following endurance exercises, athletes have elevations in serum markers of myocyte damage and reduction in diastolic and systolic function [26], although these changes return to baseline within 24 hours. In the evaluation of 45 veteran endurance athletes [23], echocardiographic evidence of left ventricular hypertrophy and elevations in serum markers of collagen synthesis, degradation and inhibition of degradation were documented, suggesting the presence of cardiac fibrosis related to long-term athletic activity. Whyte et al. [27] reported the case of a 57 year old athlete who died suddenly while running a marathon. Autopsy revealed biventricular fibrosis, left ventricular hypertrophy and reduction of left ventricular chamber volume.

## Conclusion

The true incidence and degree of age-related myocardial fibrosis is unknown; both animal and human models have documented its occurrence. Whether these age-related changes are due primarily to the diseases more frequently seen in the elderly, namely hypertension and inflammation, or arose de novo due to decreased collage breakdown is also uncertain. More recent reports of exercise-induced fibrosis raise the question of late pathological consequences of physical activity. Hypertension-related myocardial fibrosis is at least partly preventable with pharmacotherapy. Further understanding of the mechanisms associated with age- and exercise-induced fibrosis may likewise lead to the discovery of chemical pathways that can be pharmacologically altered, allowing for the prevention of cardiac dysfunction associated with fibrotic change.

## List of abbreviations

angiotensin-converting enzyme (ACE); electrocardiography (EKG); extracellular-signal-related kinase (ERK); high-density lipoprotein (HDL); Jun N-terminal kinase (JNK); low-density lipoprotein (LDL); messenger ribonucleic acid (mRNA); nicotinamide adenine dinucleotide phosphate, reduced form (NADPH); renin-angiotensin-aldosterone system (RAAS); reactive oxygen species (ROS)

## Competing interests

The author(s) declare that they have no competing interests.

## Authors' contributions

SL and LH secured the case, conducted the literature review, and participated in the preparation of the manuscript. All authors read and approved the final manuscript.

## Consent

Written informed consent was obtained from the patient for publication of this case report and accompanying images. A copy of the written consent is available for review by the Editor-in-Chief of this journal.

## References

[B1] Mukherjee D, Sen S (1990). Collagen phenotypes during development and regression of myocardial hypertrophy in spontaneously hypertensive rats. Circ Res.

[B2] Song Y, Yao Q, Zhu J, Luo B, Liang S Age-related variation in the interstitial tissues of the cardiac conduction system; and autopsy study of 230 Han Chinese. Forensic Sci Int.

[B3] Burkauskiene A, Mackiewicz Z, Virtanen I, Konttinen YT (2006). Age-related changes in myocardial nerve and collagen networks of the auricle of the right atrium. Acta Cardiol.

[B4] Villari B, Vassalli G, Schneider J, Chiariello M, Hess OM (1997). Age dependency of left ventricular diastolic function in pressure overload hypertrophy. J Am Coll Cardiol.

[B5] Klima M, Burns TR, Chopra A (1990). Myocardial fibrosis in the elderly. Arch Pathol Lab Med.

[B6] Unverferth DV, Baker PB, Swift SE, Chaffee R, Fetters JK, Uretsky BF, Thompson ME, Leier CV Extent of myocardial fibrosis and cellular hypertrophy in dilated cardiomyopathy. Am J Cardiol.

[B7] Besse S, Robert V, Assayag P, Delcayre C, Swynghedauw B (1994). Nonsynchronous changes in myocardial collagen mRNA and protein during aging: effect of DOCA-salt hypertension. Am J Physiol.

[B8] Robert V, Besse S, Sabri A, Silvestre JS, Assayag P, Nguyen VT, Swynghedauw B, Delcayre C (1997). Differential regulation of matrix metalloproteinases associated with aging and hypertension in the rat heart. Lab Invest.

[B9] Susic D, Varagic J, Frohlich ED (1999). Pharmacologic agents on cardiovascular mass, coronary dynamics and collagen in aged spontaneously hypertensive rats. J Hypertens.

[B10] Weber KT, Brilla CG (1992). Myocardial fibrosis and the renin-angiotensin-aldosterone system. J Cardiovasc Pharmacol.

[B11] Weber KT, Brilla CG, Janicki JS, Reddy HK, Campbell SE (1991). Myocardial fibrosis: role of ventricular systolic pressure, arterial hypertension, and circulating hormones. Basic Res Cardiol.

[B12] Weber KT, Sun Y, Guarda E, Katwa LC, Ratajska A, Cleutjens JP, Zhou G (1995). Myocardial fibrosis in hypertensive heart disease: an overview of potential regulatory mechanisms. Eur Heart J.

[B13] Wakatsuki T, Schlessinger J, Elson EL (2004). The biochemical response of the heart to hypertension and exercise. Trends Biochem Sci.

[B14] Nicoletti A, Michel JB (1999). Cardiac fibrosis and inflammation: interaction with hemodynamic and hormonal factors. Cardiovasc Res.

[B15] Hahn AW, Jonas U, Buhler FR, Resink TJ Activation of human peripheral monocytes by angiotensin II. FEBS Lett.

[B16] Hinglais N, Heudes D, Nicoletti A, Mandet C, Laurent M, Bariéty J, Michel JB (1994). Colocalization of myocardial fibrosis and inflammatory cells in rats. Lab Invest.

[B17] Nicoletti A, Heudes D, Mandet C, Hinglais N, Bariety J, Michel JB (1996). Inflammatory cells and myocardial fibrosis: spatial and temporal distribution in renovascular hypertensive rats. Cardiovasc Res.

[B18] Zhang GX, Lu XM, Kimura S, Nishiyama A Role of mitochondria in angiotensin II-induced reactive oxygen species and mitogen-activated protein kinase activation. Cardiovasc Res.

[B19] Cave A, Grieve D, Johar S, Zhang M, Shah AM NADPH oxidase-derived reactive oxygen species in cardiac pathophysiology. Philos Trans R Soc Lond B Biol Sci.

[B20] Thomas DP, Zimmerman SD, Hansen TR, Martin DT, McCormick RJ (2000). Collagen gene expression in rat left ventricle: interactive effect of age and exercise training. J Appl Physiol.

[B21] Maron BJ (1986). Structural features of the athlete heart as defined by echocardiography. J Am Coll Cardiol.

[B22] Maron BJ, Pelliccia A, Spataro A, Granata M (1993). Reduction in left ventricular wall thickness after deconditioning in highly trained Olympic athletes. Br Heart J.

[B23] Lindsay MM, Dunn FG (2007). Biochemical evidence of myocardial fibrosis in veteran endurance athletes. Br J Sports Med.

[B24] Miki T, Yokota Y, Seo T, Yokoyama M (1994). Echocardiographic findings in 104 professional cyclists with follow-up study. Am Heart J.

[B25] Nishimura T, Yamada Y, Kawai C (1980). Echocardiographic evaluation of long-term effects of exercise on left ventricular hypertrophy and function in professional bicyclists. Circulation.

[B26] Whyte G, George K, Shave R, Dawson E, Stephenson C, Edwards B, Gaze D, Oxborough D, Forster J, Simspon R (2005). Impact of marathon running on cardiac structure and function in recreational runners. Clin Sci (Lond).

[B27] Whyte GP, Sheppard M, George K, Shave R, Wilson M, Prasad S, O'hanlan R, Sharma S (2007). Post-mortem evidence of idiopathic left ventricular hypertrophy and idiopathic interstitial myocardial fibrosis: Is exercise the cause?. Br J Sports Med.

